# Building workforce capacity for complex care coordination: a function analysis of workflow activity

**DOI:** 10.1186/1478-4491-12-52

**Published:** 2014-09-13

**Authors:** Liza Heslop, Rebecca Power, Kathryn Cranwell

**Affiliations:** College of Health and Biomedicine, Victoria University, PO Box 14428, Melbourne, VIC 8001 Australia; Strategy, Service Planning and Partnering with Consumers, Royal Victorian Eye and Ear Hospital, 32 Gisborne St, East Melbourne, VIC 3002 Australia; Community Services Workforce Innovation and Integration Lead, Western Health, 176 Furlong Road, St Albans, VIC 3021 Australia

**Keywords:** Care coordination, Chronic disease management, Health care, Health workforce reform, Interprofessional teams, Workflow modelling

## Abstract

**Background:**

The care coordination workforce includes a range of clinicians who manage care for patients with multiple chronic conditions both within and outside a hospital, in the community, or in a patient’s home. These patients require a multi-skilled approach to support complex care and social support needs as they are typically high users of health, community, and social services. In Australia, workforce structures have not kept pace with this new and emerging workforce. The aim of the study was to develop, map, and analyse workforce functions of a care coordination team.

**Methods:**

Workflow modelling informed the development of an activity log that was used to collect workflow data in 2013 from care coordinators located within the care coordination service offered by a Local Health Network in Australia. The activity log comprised a detailed classification of care coordination functions based on two major categories – direct and indirect care. Direct care functions were grouped into eight domains. A descriptive quantitative investigation design was used for data analysis. The data was analysed using univariate descriptive statistics with results presented in tables and a figure.

**Results:**

Care coordinators spent more time (70.9%) on direct care than indirect care (29.1%). Domains of direct care that occupied the most time relative to the 38 direct care functions were ‘Assessment’ (14.1%), ‘Documentation’ (13.9%), ‘Travel time’ (6.3%), and ‘Accepting/discussing referral’ (5.7%). ‘Administration’ formed a large component of indirect care functions (14.8%), followed by ‘Travel’ (12.4%). Sub-analyses of direct care by domains revealed that a group of designated ‘core care coordination functions’ contributed to 40.6% of direct care functions.

**Conclusions:**

The modelling of care coordination functions and the descriptions of workflow activity support local development of care coordination capacity and workforce capability through extensive practice redesigns.

## Background

Care coordination is an umbrella term used for a range of health care roles that offer seamless support to patients who have complex health care, community, and social support needs [1]. There is no one accepted definition of care coordination [2]; its implementation follows diverse rules, regulations, guidelines, and service models [3, 4]. Despite it being a concept that requires clarification [5–7], health care organisations have increased investment in care coordination service models to improve health outcomes for patients with complex chronic care and social support needs and to reduce the burden and complications of hospital re-admissions. To meet the needs of this growing patient group, new care coordination roles that traverse generalist and specialist clinical roles have burgeoned. Care coordination workforce reform and innovation have been supported by international initiatives, particularly in the US, where most states have standards, guidelines, and state laws to determine who may provide such services, as well as credentialing and licensing requirements to ensure those recruited to the roles are qualified [8].

In Australia there is no statutory framework to govern the care coordination workforce; there are no professional guidelines, consistent care type definitions or business rules to support care coordination. Various care coordination service models reported within the Australian literature tend to have been initiated by, and form part of, the authorising environment of the Australian Primary Health Care (PHC) system. The models mostly involve, or are led by, general medical practitioners as part of General Practice clinics, Aboriginal Health Services and Medicare Locals [9, 10], while other models form part of the Australian Primary Mental Health Care system [11].

The current study focuses on a care coordination service which is administered by a Local Health Network (LHN) while also being part of a public hospital. LHNs provide public hospital services in Australia and can contain one or more hospitals as part of a business group, geographical area, or community; they receive activity-based and block funding under the National Health Reform Agreement [12]. Thus, the care coordination service does not sit under PHC or Home and Community Care funding models. For the purposes of setting the care coordination context for this study, care coordination is defined as the provision of ‘non-admitted’ health care and support services to patients with complex and chronic health care conditions, by a public hospital. ‘Non-admitted’ care is a classification of activity-based policy and funding in Australia and includes Tier 2 services provided by hospitals to patients who do not undergo formal admission and do not occupy a hospital bed [13]. All care coordination activities of the LHN, including staffing and expenditure, are mostly reported for inclusion in the national ‘non-admitted’ activity-based dataset; however, the national model is still being refined and tested with regard to ‘non-admitted’ services.

In order to improve service delivery and the continuity between service providers across regional partners in primary, community, and acute settings, and to relieve pressure on the region’s acute in-patient hospital services, the LHN developed a vision and strategy to help shape a responsive care coordination model of service to meet the complex health care needs of the population it serves in the Western region of Melbourne, Australia. Care coordination designates services where providers and clients work closely together to achieve common patient-centred goals of care. It forms an integral part of the concept of care continuity where there can be incomplete knowledge of providers’ actions to meet clients’ goals of care [14].

Under the LHN model, care coordination requires that discharge planning links the primary and community care services that are delivered by an integrated interprofessional team consisting of allied health practitioners, nurses, and support workers. This collaborative approach ensures that patients’ health care requirements are planned and delivered in a logical, connected, and timely manner. It may include activities traditionally classified as case management such as arranging additional services through sub-contracting or purchase of services, the maintenance of agreements between organisations, organising case conferences, and the active monitoring of patient care plans with due consideration to family circumstances. In this service, not all referrals arise from an inpatient admission.

The care coordination service model at the LHN includes five specialist care coordination services known as the Immediate Response Service, Hospital Admission Risk Program, Aged Care Assessment Service, Post-Acute Care service, and the community-based Transitional Care Program. The Hospital Admission Risk Program service has been reported to be an effective service integration model as it reduced demand for hospital admissions without incurring cost [15]. As an umbrella for services the LHN has, over time, attracted a substantive care coordination workforce providing generalist and specialist clinical and non-clinical roles. Despite this, internal care coordination workforce planning frameworks and mechanisms to build workforce capability were found to be insufficiently detailed. It was essential for the LHN to focus on developing high performing, interprofessional teams, capable of working flexibly across professional boundaries to optimise care management, care coordination, inter-sectorial linkages, and planning. In order to reconfigure care coordination roles and support those roles with general capability and career pathways, the LHN required information about the nature of interprofessional care coordination activity and the distribution of care coordinators’ functions within the care coordination service.

The aim of this study was to map and analyse the workflow functions of an existing care coordination workforce whose sole role was within a care coordination service. All the functions and activities performed by the care coordination workforce were identified and mapped into a model or classification of care coordination workforce functions and the time taken for each activity according to function and domain was quantified.

## Methods

### Design

A descriptive quantitative design informed the overall approach to workflow function analysis. Workflow functions are all the tasks performed by care coordinators in a given day. Workflow modelling is a linear task-oriented approach that can be used to represent the day-to-day performance of workflow functions and to assist with work process redesign [16]. Conceptually, we have followed Davenport’s [17] definition of workflow process as a specific ordering of work activities across time, with a beginning and an end. Workflow modelling designs with wide-ranging applications to many industries have been systematically researched and developed into a Workflow Elements Model to assist researchers determine elements implicit to applied workflow modelling [18]. The model provides various elements associated with different ‘motivational’ and ‘methodological’ orientations. This study adopts two elements from the model described as ‘temporality’, which relates to the relationships among routine tasks, and ‘time study’ where the analysis shows how much time individual functions or activities contribute to workflow [18].

Workflow modelling methods have been applied previously in health care studies to describe work patterns of personal care workers in nursing homes [19], the activity of dieticians [20], and the care coordination activity of primary care nurses [21]. They have been used to build workforce flexibility, productivity, and efficiency [22]; a Canadian workflow modelling study generated outcomes that were used to support nursing role restructure across 17 acute health sites in British Columbia [23]. This study applies workflow modelling methods to represent and describe the components of workflow functions of an interprofessional service providing complex care coordination.

### The setting

The setting for this study is a LHN in Victoria, Australia – a major regional public provider of acute and sub-acute health services throughout Western metropolitan Melbourne. The population of the Western region of Melbourne is approximately 823,400 people; it will be one of Australia’s fastest growing areas with a predicted population growth of 23% by the end of 2021 relative to 2011 population levels [24]. The region has a high proportion of culturally and linguistically diverse communities where English may not be the first language and where the population comprises a higher burden of disease than national averages, combined with a high proportion of socio-economic disadvantage [25]. The LHN also delivers a comprehensive suite of medication support services, a range of community services supporting discharge, rehabilitation, and recovery, and programs offered in conjunction with community partners which support people with complex and chronic care needs.

The LHN’s care coordination workforce comprises clinical staff from four health care disciplines – nurses, social workers, physiotherapists, and occupational therapists. In addition, the service includes one support worker designated as an allied health assistant. The breakdown of the total full-time equivalent staff of 87.15 for 2013 was: Nursing (professional) (53.5); Allied Health (30.25); Psychology/pharmacy (2.4); Allied Health Assistant (1).

### Participants

Participants in the study comprised all care coordinators rostered on duty during a two-day period of data collection. The study received ethical approval from relevant human research ethics committees (Victoria University Human Research Ethics Committee & Western Health Human Research Ethics Committee). Informed consent was obtained from all participants.

### Instrument activity log

A workflow activity log with considerable detail of all the LHN’s care coordination workforce functions was developed into a spreadsheet and used to collect data. Care coordination literature on care coordinators mandated work functions was sourced and reviewed to assist with the descriptions of direct care functions and the eight direct care function domains [26–28]. The resultant workflow classifications of care coordinators’ functions were determined to suit the activity of the setting’s context.

Development and pre-testing of the activity log was conducted at two levels. Firstly, a clinical reference group of care coordination clinicians assisted with the development and review of the activity log. The reference group comprised six senior clinicians who had five or more years’ experience in care coordination. Secondly, ten care coordinators trialled the activity log on-site to ensure all components of workflow were captured. Results from the trial resulted in several modifications to the classification of functions and the activity log. The trial highlighted the need for adjustment to the initially proposed 15 minute data collection periods as care coordinators frequently switched between direct and indirect care functions, in periods under 15 minutes. In the trial, extensive efforts were made to ensure care coordinators were able to allocate a workflow process to each category in a consistent manner despite the fact that each workflow process can present differently in certain circumstances. These efforts resulted in a detailed spreadsheet – the activity log – that was used as the data collection tool.

Workflow functions listed on the activity log were classified into two major categories for recording. These were denoted as direct care (patient attributable), comprising 38 functions, and indirect care (non-patient attributable), comprising 24 functions. Direct care refers to care coordinators’ workflow activities that specifically relate to meeting patients’ needs. Indirect care refers to care coordinators’ activities that focused on maintaining the environment in which care is delivered. The fine-tuning between these two categories was supported by the trial and clinical reference group review.

Comprehensive instructions and guidelines were given to each participant and field-based support was provided to ensure shared understandings of key terms and dependable data entry. Full descriptions of each function were provided to the participants to assist with accurate self-recordings in the activity log. Activity logs were collected for two consecutive days. There were 53 care coordinators rostered on duty over the two day period and a total of 106 logs were returned.

### Data collection, management, and analysis

All care coordinators on duty for the two day period were provided with hard copies of the activity log in the spreadsheet format for recording their time against each function.

The data from the activity logs were loaded into a spreadsheet and were then analysed using SPPS Version 20.0 for univariate descriptive statistics. When recording workflow data, care coordinators largely accounted for their activity in 15 minute periods. When different functions occurred during the 15 minute period, these were recorded within 5 minute periods. Based on the raw data of all functions recorded, the total time recorded in minutes, the mean time recorded in minutes and standard deviation of the mean and the percentage of total time attributed to each function were calculated.

In order to improve data management for analysis, the clinical reference group and research team reviewed the preliminary analysis of data and further categorisation of direct care functions was undertaken. Direct care functions were noted to occupy more than two thirds of workflow activity, representing a large and clinically meaningful group of activity. All 38 direct care functions were classified into 8 major domains to represent key areas of direct care workflow, namely: ‘Access’, ‘Assessment’, ‘Providing consultation’, ‘Arranging care’, ‘Contracting’, ‘Treatment’, ‘Preparation/administration’, and ‘Other’. For example, the functions designated as ‘Active case finding’, ‘Accepting/discussing referral’, and ‘Prioritising patients’ were categorised within the direct care function domain ‘Access’. The domains are simply a subset to represent groupings of direct care functions. Some examples of indirect care functions include ‘Administration’, ‘Time-out break’, and ‘Budget/financial’.

## Results

The total number of functions recorded was n_1_ = 3,520. Of these, 2,637 (n_2_) were direct care functions and 875 (n_3_) were indirect care functions. There were 8 missing functions (0.2%). The total work time recorded over the two-day period was 54,540 minutes (90.9 hours). Comparisons between time expended on direct and indirect care functions (Table 1) shows the proportion of total time spent on direct care functions (70.9%) was more than two thirds of the total time spent on indirect care functions (29.1%).

**Table 1:** **Comparisons between direct and indirect care functions**

Function	n _1_ = 3,520	Total time (minutes)	Mean time (minutes) (standard deviation)	Percentage of total time attributed to each function
Direct care	n_2_ = 2,637	38,695	14.6 (17.0)	70.9%
Indirect care	n_3_ = 875	15,845	18.1 (16.1)	29.1%
Total	3,512	54,540	15.5 (16.9)	100%

Analysis of direct care functions (Table 2) reveals that the highest proportion of time for direct care activity, relative to all time spent on direct care activity, was spent on ‘Assessment’ (function 2.2, 14.1%) and ‘Documentation’ (function 7.4, 13.9%). The lowest proportion of time was spent on ‘Patient complaints’ (function 6.7, 0.05%), followed by ‘Working with interpreters’ (function 7.1, 0.1%), ‘Diversion from admission’ (function 6.2, 0.4%), ‘Equipment – prescription/provision’ (function 6.4, 0.4%), ‘Liaison – Psychiatry’ (function 4.2, 0.5%), ‘Negotiating/advocacy’ (function 4.8, 0.5%), and ‘Handover’ (function 4.7, 0.5%).

**Table 2:** **Descriptive analysis for direct care functions**

Function number and name		Functions recorded n _2_ = 2637	Total time (minutes)	Mean time (minutes) (standard deviation)		Percentage of total time attributed to each function	Rank of percentage of total time attributed to the function
1.1	Active case finding	61	990	16.2	(12.6)	2.6%	14
1.2	Accepting/discussing referral	230	2,195	9.5	(6.2)	5.7%	4
1.3	Prioritising patients	30	310	10.3	(6.1)	0.8%	28
2.1	Risk screening	47	675	14.4	(10.0)	1.7%	19
2.2	Assessment	209	5,440	26.0	(24.7)	14.1%	1
2.3	Assessment – other specialised	47	1,390	29.6	(27.2)	3.6%	7
3.1	Community	22	400	18.2	(18.9)	1.0%	26
3.2	Medical staff	17	245	14.4	(14.4)	0.6%	31
3.3	Nursing staff	35	635	18.1	(18.1)	1.6%	22
3.4	Other (comment)	56	1,100	19.6	(20.3)	2.8%	12
4.1	Liaison – Medical/nursing staff	99	940	9.5	(5.3)	2.4%	16
4.2	Liaison – Psychiatry	14	175	12.5	(8.4)	0.5%	32
4.3	Liaison – Community agencies	72	745	10.3	(8.1)	1.9%	18
4.4	Liaison – General Practitioner	23	265	11.5	(6.9)	0.7%	30
4.5	Liaison – Other	111	1,050	9.5	(7.3)	2.7%	13
4.6	Meeting (e.g., ward, unit)	27	1,475	54.6	(31.6)	3.8%	6
4.7	Handover	21	200	9.5	(4.1)	0.5%	33
4.8	Negotiating/advocacy	19	195	10.3	(7.7)	0.5%	34
4.9	Patient monitoring	105	1,170	11.1	(8.7)	3.0%	11
4.10	Referral – internal	26	315	12.1	(9.7)	0.8%	29
4.11	Referral – external	102	1,260	12.4	(9.1)	3.3%	9
5.1	Contracting/liaising with provider – internal	65	495	7.6	(4.7)	1.3%	25
5.2	Contracting/liaising with provider – external	192	1,560	8.1	(11.7)	4.0%	5
6.1	Education	80	1,310	16.4	(17.0)	3.4%	8
6.2	Diversion from admission	17	150	8.8	(7.4)	0.4%	35
6.3	Counselling /health coaching	42	665	15.8	(8.1)	1.7%	20
6.4	Equipment – prescription/provision	9	160	17.8	(7.9)	0.4%	36
6.5	Follow-up call after discharge	47	340	7.2	(3.5)	0.9%	27
6.6	Carer support	43	580	13.5	(7.7)	1.5%	23
6.7	Patient complaints	2	20	10.0	(0.0)	0.05%	38
6.8	Technical-clinical care	25	915	36.6	(70.3)	2.4%	17
7.1	Working with interpreters	4	50	12.5	(2.8)	0.1%	37
7.2	Reading medical history/report	92	1,285	14.0	(10.0)	3.3%	10
7.3	Background research	52	655	12.6	(9.4)	1.7%	21
7.4	Documentation	353	5,380	15.2	(16.9)	13.9%	2
7.5	Set up/clean up	36	585	16.3	(18.2)	1.5%	24
7.6	Travel time (including walking)	129	2,420	18.8	(10.9)	6.3%	3
8.1	Other (comment)	76	955	12.6	(17.9)	2.5%	15
Total		2637	38,695	14.6	(17.0)	100.0%	

Within the direct care functions, variation (standard deviation; SD) for function duration was highest for ‘Technical-clinical care’ (function 6.8, SD = 70.3), ‘Meeting (e.g., ward, unit)’ (function 4.6, SD = 31.6), and ‘Assessment – other specialised’ (function 2.3, SD = 27.2) compared to the variation for all direct care functions (n_2_ = 2,637) SD =17.0. For these direct care functions, the time spent by individual care coordinators varied greatly. Such variation may be attributed to differences within the five specialist care coordination services that make up the service and/or there may have been differences across the four professional groups. Those functions with the lowest variation (SD), excluding ‘Patient complaints’ and ‘Working with interpreters’ where only 2 and 4 functions were recorded, respectively, namely – ‘Follow-up call after discharge’ (function 6.5, SD = 3.5), ‘Handover’ (function 4.7, SD = 4.1), and ‘Liaison – Medical/nursing staff’ (function 4.1, SD = 5.3) may be considered to be more standardised and similar with regard to time expenditure.

Analysis of direct care functions by domain (Tables 3) reveals the ‘Preparation/administration’ function domain was the most time-consuming of all the function domains (26.8%).

**Table 3:** **Descriptive analysis of direct care functions by domain**

Function domain number and name		Functions recorded n _2_ = 2,637	Total time (minutes)	Mean time (minutes) (standard deviation)		Percentage of total time attributed to each function domain
1	Access	321	3,495	10.9	(8.2)	9.0%
2	Assessment	303	7,505	24.8	(23.8)	19.4%
3	Providing consultation	130	2,380	18.3	(18.7)	6.2%
4	Arranging care	619	7,790	12.6	(13.4)	20.1%
5	Contracting	257	2,055	8.0	(10.4)	5.3%
6	Treatment	265	4,140	15.6	(24.9)	10.7%
7	Preparation/administration	666	10,375	15.6	(14.7)	26.8%
8	Other	76	955	12.6	(17.9)	2.5%
Total		2,637	38,695	14.6	(17.0)	100.0%
**Direct care domain**		**Function and number**				
1. Access		Active case finding			1.1	
		Accepting/discussing referral			1.2	
		Prioritising patients			1.3	
2. Assessment		Risk screening			2.1	
		Assessment			2.2	
		Assessment – other specialised			2.3	
3. Providing consultation		Community			3.1	
		Medical staff			3.2	
		Nursing staff			3.3	
		Other (comment)			3.4	
4. Arranging care		Liaison – Medical/nursing staff			4.1	
		Liaison – Psychiatry			4.2	
		Liaison – Community agencies			4.3	
		Liaison – General Practitioner			4.4	
		Liaison – Other			4.5	
		Meeting (e.g., ward, unit)			4.6	
		Handover			4.7	
		Negotiating/advocacy			4.8	
		Patient monitoring			4.9	
		Referral – internal			4.10	
		Referral – external			4.11	
5. Contracting		Contracting/liaising with provider – internal			5.1	
		Contracting/liaising with provider – external			5.2	
6. Treatment		Education			6.1	
		Diversion from admission			6.2	
		Counselling/health coaching			6.3	
		Equipment – prescription/provision			6.4	
		Follow-up call after discharge			6.5	
		Carer support			6.6	
		Patient complaints			6.7	
		Technical-clinical care			6.8	
7. Preparation/administration		Working with interpreters			7.1	
		Reading medical history/report			7.2	
		Background research			7.3	
		Documentation			7.4	
		Set up/clean up			7.5	
		Travel time (including walking)			7.6	
8. Other		Other (comment)			8.1	

This is partly attributable to the high percentage of time recorded for ‘Documentation’ (13.9%) within this domain (Table 2). The function domains of ‘Arranging care’ (20.1%) and ‘Assessment’ (19.4%) also required significant time expenditure. Analysis of indirect care functions (Table 4), excluding ‘Time-out break’ and ‘Time completing the activity log’, shows the most time-consuming functions were ‘Administration’ (14.8%), ‘Travel’ (12.4%), and ‘Meetings’ (8.0%).

**Table 4:** **Descriptive analysis of indirect care functions**

Function number and name		Functions recorded n _3_ = 875	Total time (minutes)	Mean time (minutes) (standard deviation)		Percentage of total time attributed to each function	Rank of percentage of total time attributed to the function
1	Administration	194	2,350	12.1	(8.2)	14.8%	2
2	Time-out break	164	3,290	20.1	(11.8)	20.8%	1
3	Budget/financial	1	20	20.0	(0.0)	0.1%	22
4	Consultation (non-clinical)	39	405	10.4	(5.6)	2.6%	9
5	Human resources, e.g., appraisal, staff support	4	110	27.5	(15.5)	0.7%	17
6	Human resources – Recruitment	0	0	0.0	(0.0)	0.0%	Not ranked
7	Informal discussion with peers	35	380	10.9	(5.4)	2.4%	10
8	Meetings	41	1,260	30.7	(21.9)	8.0%	5
9	Orientation	3	95	31.7	(12.5)	0.6%	19
10	Planning	38	980	25.8	(34.3)	6.2%	6
11	Presentations/inservices	0	0	0.0	(0.0)	0.0%	Not ranked
12	Professional development	7	240	34.3	(20.2)	1.5%	13
13	Quality improvement activity	21	865	41.2	(38.2)	5.5%	7
14	Report writing/evaluation	4	135	33.8	(18.8)	0.9%	16
15	Research	7	110	15.7	(6.0)	0.7%	18
16	Rostering/work allocation	11	365	33.2	(27.5)	2.3%	11
17	Service development	2	85	42.5	(24.7)	0.5%	20
18	Statistics	35	665	19.0	(14.6)	4.2%	8
19	Supervision/mentoring (provider)	11	225	20.5	(10.5)	1.4%	14
20	Supervision/mentoring (recipient)	2	35	17.5	(17.6)	0.2%	21
21	Teaching (students)	12	160	13.3	(9.3)	1.0%	15
22	Time completing the activity log	127	1,810	14.3	(11.2)	11.4%	4
23	Travel (including walking)	96	1,960	20.4	(11.6)	12.40%	3
24	Other	21	300	14.3	(13.5)	1.9%	12
Total		875	15,845	18.1	(16.1)	100.0%	

### Sub-analyses of selected domains of direct care activity

Three groupings of direct care domains were also established by the clinical reference group and research team. This was done in order to represent the data in an administratively meaningful way. These groups were designated ‘Assessment and treatment’, ‘Clinical administration’, and ‘Core care coordination functions’. The group ‘Assessment and treatment’ included direct care domain numbers 2 and 6 (‘Assessment’ and ‘Treatment’), ‘Clinical Administration’ included the domain numbers 7 and 8 (‘Preparation/Administration’ and ‘Other’), and ‘Core care coordination functions’ included direct care domain numbers 1, 3, 4, and 5 (‘Access’, ‘Providing Consultation’, ‘Arranging Care’, and ‘Contracting’).

**Figure 1:**
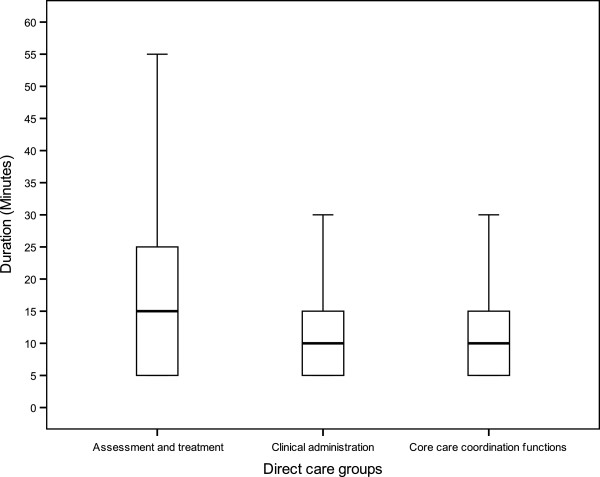
**Box plots for time duration for three groups of direct care functions.**

‘Core care coordination functions’ were shown to contribute more to overall function time durations than the other two groups ‘Assessment and treatment’ and ‘Clinical Administration’ (40.6% vs. 30.1% and 29.3%, respectively; Table 5).Figure 1 depicts the box plots of time duration for these three groups using interquartile ranges. Note that the 25th percentile is equal to the minimum categorical values of time duration where the minimum duration recorded was 5 minutes. As illustrated in Figure 1, there is a smaller interquartile range for ‘Core care coordination functions’ and ‘Clinical administration’ (Q3 – Q1 = 15 – 5 = 10 minutes), respectively, versus ‘Assessment and treatment’ (Q3 – Q1 = 25 – 5 = 20 minutes). Consistent with the above observation, the group ‘Core care coordination functions’ has less variation of function time duration (SD = 12.8) in comparison to ‘Clinical administration’ (SD = 15.1) and ‘Assessment and treatment’ (SD = 24.8). The smaller SD value for functions implies that these functions have less time duration variations; hence, these functions are more stable and predictable in time required even when performed by staff in different situations and across different services and professional groups.

**Table 5:** **Sub-analysis of direct care domain groupings**

Domain Group	Domain	Percentage of total time attributed to each function domain
Assessment and treatment	Assessment	19.4%
	Treatment	10.7%
**(Total for group)**		**(30.1%)**
Clinical administration	Preparation/administration	26.8%
	Other	2.5%
**(Total for group)**		**(29.3%)**
Core care coordination functions	Access	9.0%
	Providing consultation	6.2%
	Arranging care	20.1%
	Contracting	5.3%
**(Total for group)**		**(40.6%)**
**Total time for direct care functions**		**100%**

## Discussion

To our knowledge, there is no descriptive quantitative care coordination workflow process study available based on an interprofessional workforce providing services for patients with chronic illness and complex needs. Further, there are no studies available using workflow modelling to enable reliable comparisons in the interprofessional context we have described. The field of care coordination has been described as ‘immature’ because of the inherent challenges around building consensus on ‘what constitutes care coordination’, as well as the need to advance the systematic study of care coordination to build an evidence base inclusive of care coordination measurement [3]. Published studies that share some similarities to the methods of the current study include a US observational study of the work activity of ten primary care nurses undertaken to help understand the roles and tasks carried out by nurses in primary care, reporting that only 15% of total nurse work activity was ‘care coordination’ [21]. Findings from a health care workflow study undertaken with dieticians in three Australian hospitals which investigated designated workflow categories of direct and indirect care revealed the percentage of time spent on direct and indirect patient support activities was 18.3% and 40.4%, respectively [20]. In contrast, our results show that direct care functions occupied more than two thirds of all care coordination activity in the context we studied.

Despite the emerging landscape concerning the science of care coordination, research has clarified and determined key concepts about workflow processes of interprofessional care which are also very dependent on context. An Australian study that more or less sits within the general practice model mentioned previously [9], offers a qualitative investigation into core processes that define the work of care coordinators, illuminating understandings of care coordination and its unique features for this context [29]. Participants were care coordinators comprising ten general practitioners and six registered nurses who delivered care coordination as members of a general practice team. Thematic analysis of participants’ interviews revealed four distinguishing themes: ‘moving beyond usual practice by spanning boundaries’; ‘relationship-based care’; ‘agreed roles and routines among relevant parties’; and ‘committing to chronic condition care coordination’ [29]. The study points to attributes and relational features of care coordination involving multiple disciplines and stakeholder groups. The concept of ‘relational processes’ in care coordination that involve complementary roles of each professional and their interdependencies have been identified as important processes in emerging interprofessional teams where the building of ‘relational coordination’ may ensure team functioning based upon shared goals, shared knowledge, and mutual respect [30].

### Study limitations, strengths, and future research

This study was based upon a service model of care coordination where the context itself and the interactions between the contextual elements, the interprofessional configuration, and work functions are unique. Still, the service model does share similarities with international strategies and developments in care coordination models where non-physician providers are leading interprofessional team-based models to support care coordination in health systems [5]. Even so, differences in work functions according to context suggests that direct application of the classification proposed in this study to a range of care coordination models, particularly in PHC, may not be suitable. Another limitation is the modest two-day sampling, though every effort was made within research procedures to ensure accuracy of data entry to enable contextually-reliable results, it is possible for some misrepresentation of functions to have occurred.

The strengths of this study lie in the development and granularity of the data collection instrument that offers a foundation for LHN care coordination workflow classification and the collation and description of a large number of workflow functions depicting all activity of a care coordination workforce. Knowledge of the inherent activity of this workforce and the amount of time spent on different workflow functions provides a robust evidence-based resource for policy development concerning interprofessional service approaches. Measures of workflow functions captured in this study will be useful to Australian policy officials to support refinement and development of future revisions to the counting and funding rules for Tier 2 non-admitted services. Workflow function variables have been based on the type of clinician providing the service which can differentiate one aspect of resource utilisation for non-admitted services. Nevertheless, workflow must be considered as a dynamic process and some of the functions explicated in the instrument had very short time duration. This suggests the need for further research to refine and test the classifications used in the study’s instrument. Further research may be generated from this study to estimate costs associated with conducting care coordination activity, including cross-service comparisons. Future research must also consider the impact of interprofessional workforce models on clinical and economic outcomes.

## Conclusions

The capacity of this LHN to provide high quality care coordination services to the rapidly expanding Western region of Melbourne, Australia, where the risks associated with low health and literacy levels are coupled with a high proportion of chronic illness, has unquestionably required prompt and effective innovation to develop capability and efficiency of a care coordination workforce. The findings of this study have provided valuable baseline descriptive workforce information about the workflow activity of the LHN’s care coordination workforce that is supporting ongoing organised workforce reform and from which future change to this workforce can be monitored. The reform strategies currently in progress include care coordination role reconfiguration with unique fit-for-purpose roles and defined career pathways. The care coordination workforce reform vision, currently being enacted, is to improve the balance between specialist and generalist roles, establish specialist generalist roles with expanded scopes of practice, develop standards, tools, and protocols, and education and training.

In Australia, national activity-based funding policies are being developed to support multi-disciplinary ‘clinic’ models that currently fit within the Australian Tier 2 non-admitted care framework [31]. As high volumes of non-admitted services are provided in Australia, the Independent Hospital Pricing Authority intends to progress, in the longer term, the development of a ‘non-admitted services’ classification that can support activity-based funding [31]. Activity-based policy concerning funding and performance for ‘non-admitted services’ may be improved with the understanding of care coordination activities that this paper has offered. Multidisciplinary care models of a ‘clinic’ service may not be appropriately developed in the existing Australian non-admitted Tier 2 classifications system. Given the expected growth in usage of non-admitted care services, further analysis of care coordination service activity deserves attention. Managers of health services may also consider options for building workforce capacity to manage chronic and complex illness, where roles can be configured within non-admitted services that may not necessarily be dependent on physician stewardship but where nurses and allied health professionals as non-physician providers play a central role. Policy approaches that create operational partnerships between LHNs, community-based health services, and PHC services are needed to better deliver services to patients with the most complex health care needs.

## Abbreviations

LHN: Local Health Network
PHC: Primary Health Care.
